# Predictors of response to lenvatinib in advanced differentiated thyroid cancer: focus on the CONUT score

**DOI:** 10.1007/s12020-025-04228-5

**Published:** 2025-04-11

**Authors:** Edoardo Talpacci, Silvia Morelli, Vincenzo Leone, Sonia Moretti, Miriam Paci, Vittorio Bini, Efisio Puxeddu

**Affiliations:** Section of Internal Medicine and Endocrine and Metabolic Sciences, Department of Medicine and Surgery, University of Perugia, Perugia, Italy

**Keywords:** Progressive radioiodine-refractory differentiated thyroid cancer, Lenvatinib, CONUT score, Progression free survival

## Abstract

**Objective:**

Lenvatinib is a multityrosine kinase inhibitor approved for progressive radioiodine refractory differentiated thyroid cancer (RAI-R-DTC). Despite its efficacy, most of the initial experiences showed global inferior results if compared with SELECT study. Baseline disease stages, previous systemic treatments and baseline patients’ characteristics may affect response to therapy. The aim of our study was to review relevant clinical outcomes, identifying survival predictors, of a single center cohort of patients with advanced thyroid cancer treated with Lenvatinib.

**Methods:**

Twenty-two patients with progressive RAIR-R-DTC treated with Lenvatinib were retrospectively included. For each patient, we reviewed the main clinical baseline characteristics, including nutritional status. We evaluated the latter by using CONtrolling NUTritional status (CONUT) score. Clinical outcomes were overall survival (OS) and progression free survival (PFS).

**Results:**

At the time of analysis, 14 patients (63.6%) were alive with a median OS of 54 months (95% CI 20.35–87.65 months). Progression occurred in 16 patients (72.7%) with a median PFS of 21 months (95% CI 0–47.33 months). Nineteen out of 22 patients (86.4%) presented at least one adverse event (AE) of any grade. Baseline lower CONUT score significantly correlated with both longer PFS (HR 2.77- 95% CI 1.216–6.307, *p* = 0.015) and longer OS (HR 4.455–95% CI 1.211–16.391, *p* = 0.025). A significant longer survival was observed in patients with ECOG 0 compared to those with ECOG 1 and in the latter compared to ECOG 2 group (Log-rank test: *p* = 0.040). Conversely, higher baseline ECOG Performance Status (PS) correlated with higher severity of AEs (rs 0.468, *p* = 0.027). Finally, responder patients showed a significantly better PFS (HR 2.337–95% CI 1.091–5.006, *p* = 0.029).

**Conclusion:**

We confirmed the good efficacy of Lenvatinib treatment in progressive advanced RAI-R-DTCs showing the prognostic value of best tumor response, ECOG PS and CONUT score.

## Introduction

Differentiated thyroid cancer (DTC) accounts for about 95% of thyroid tumors and mostly shows a favorable outcome. However, less than 10% of all DTC patients develops distant metastases and, in this subset, 60–70% of them will become radio-iodine refractory, resulting in a worsening prognosis [1]. In patients with progressive radioiodine refractory differentiated thyroid cancer (RAI-R-DTC), systemic therapy with multi-kinase inhibitors (MKIs) has proven to be effective. Lenvatinib is a multiple tyrosine kinase receptor inhibitor that inhibits the kinase activities of vascular endothelial growth factor (VEGF) receptors VEGFR1 (FLT1), VEGFR2 (KDR), and VEGFR3 (FLT4), fibroblast growth factor (FGF) receptors FGFR1–4, the platelet derived growth factor receptor alpha (PDGFRα), KIT, and RET [2]. It was approved in the US in February 2015 and in the EU in May 2015 for the treatment of locally advanced or metastatic, progressive RAI-R-DTC, based on the results of the registration study SELECT [3]. In this phase III, randomized, double blinded study, Lenvatinib significantly prolonged progression free survival (PFS) compared to placebo (medians: 18.3 months vs 3.6 months; *p* < 0.001) and, according to the RECIST criteria, it was associated with an overall response rate (ORR) of 64.7% compared to 1.5% of the placebo group [3]. Furthermore, an updated analysis with a longer surveillance period confirmed the prolonged median PFS in Lenvatinib group (19.1 months) versus the placebo one (3.7 months) and showed a median PFS of 33.1 months in patients with complete or partial response compared with 7.9 months of the non-responders [4].

Despite their efficacy, MKIs are frequently associated with adverse events (AEs). Gastrointestinal side effects, anorexia, fatigue, and consequent weight loss frequently occur and they may contribute to a malnutrition state [3, 5–7]. Furthermore, disease-related malnutrition is highly prevalent among cancer patients and is associated with negative outcomes. Thus, baseline nutritional screening, aimed to improve the nutritional status, may prevent the negative consequences of malnutrition and is likely to improve overall prognosis [8, 9]. CONtrolling NUTritional status (CONUT) is an immuno-nutritional score easily applicable because based on blood parameters: albumin, lymphocyte count and total cholesterol [10, 11]. It was firstly introduced to obtain daily evaluations of inpatients undergoing routine analysis and then validated as prognostic factor in several chronic diseases, such as heart failure or end-stage liver diseases, but also in malignancies including advanced thyroid cancer [12–16].

In this study, we retrospectively reviewed relevant clinical outcomes and identified survival predictors of a RAI-R-DTC patient cohort treated with Lenvatinib.

## Materials and methods

### Study design and patients

In this monocentric study, we retrospectively evaluated the clinical charts of 27 patients suffering from progressive, locally advanced or metastatic, RAI-R DTC, treated with Lenvatinib therapy at our Institution between May 2015 and September 2023.

Patients with unavailable biochemical data concerning albumin, total cholesterol and lymphocyte count were excluded from the study.

The final population included 22 patients. For each patient, the collected baseline characteristics included age, gender, body mass index (BMI) or, whenever possible, bioelectrical impedance analysis, previous TKI systemic therapies, initial and mean Lenvatinib dose, treatment-related adverse events (AEs), histological subtype and disease stage. Furthermore, basal performance status according to Eastern Cooperative Oncology Group (ECOG) scale and basal immune-nutritional status according to CONUT score were evaluated. Written consent has been obtained from each patient. The study was approved by Regional Ethical Committee - CET Umbria (N: 4703/24).

### Efficacy assessment and clinical outcomes

Treatment efficacy was assessed by computed tomography (CT) or magnetic resonance (MRI) imaging performed every 3–6 months and tumor response was defined according to the revised RECIST guidelines (version 1.1) [17]. Whenever possible, we evaluated the best tumor response (BTR), defined as the best response recorded from the start of the treatment until disease progression. Clinical outcomes of the study were PFS and OS. The former was defined as the time from Lenvatinib start to the first evidence of tumor progression or death and the latter as the time between Lenvatinib start and death.

### CONUT score calculation

CONUT score was computed from serum albumin, lymphocyte count and total cholesterol as originally stated (Table 1) [10]. The latter were obtained by using basal blood analyses collected before Lenvatinib start. Based on the CONUT score, patients were divided into three groups:

- Group A: 0–1 (normal nutritional status);
- Group B: 2–4 (light malnutrition);
- Group C: >4 (moderate/severe malnutrition).

**Table 1:** CONUT score calculation

Serum albumin (g/dl)	>3.5	3.00–3.49	2.50–2.99	<2.5
Score	0	2	4	6
Cholesterol (mg/dl)	>180	140–179	100–139	<100
Score	0	1	2	3
Lymphocyte count (/mL)	>1600	1200–1599	800–1199	<800
Score	0	1	2	3
CONUT score	0–1	2–4	5–8	9–12
	**Normal**	**Light Malnutrition**	**Moderate Malnutrition**	**Severe Malnutrition**

### Statistical analysis

Descriptive statistics was performed to calculate patient demographic and prognostic factors.

Survival curves were calculated using the Kaplan-Meier product-limit method followed by log-rank test to evaluate differences in expected event probability between groups. To examine the risk factors affecting the prognosis, univariate and multivariate Cox proportional-hazard regression models were fitted incorporating in the multivariate model as explanatory variables all the variables that showed a significant p-value in univariate analysis. To decrease the overfit bias and internally validate our results, all univariate and multivariate regressions were subjected to 200 bootstrap resamples. Hazard ratios (HR) with 95% confidence intervals were also calculated. Correlations were checked with Spearman’s rho correlation coefficient.

Statistical analysis was performed using IBM-SPSS® version 26.0 (IBM Corp., Armonk, NY, USA, 2019). In all analyses, a two-sided *p*-value < 0.05 was considered significant.

## Results

### Study population characteristics

At baseline, median age was 68 years (41–84 years), with 3 patients aged between 40–50 years and the remaining patients older than 55 years. Median BMI was 26.9 kg/m^2^ (20.8–33.5 kg/m^2^) and 14/ 22 patients (63.6%) underwent baseline bioelectrical impedance analysis showing a median phase angle of 5.25 (3.7–7.2). Histological diagnosis was papillary thyroid carcinoma, follicular thyroid carcinoma, oncocytic thyroid carcinoma and poorly differentiated thyroid carcinoma in 10 (45.4%), 4 (18.2%), 6 (27.3%) and 2 patients (9.1%), respectively. All but one were TKI-naïve patients. ECOG performance status (PS) was 0 in 12 patients (54.5%), 1 in 9 patients (40.9%) and 2 in 1 patient (4.5%). The CONUT score was 0–1 (Group A) in 14 patients (63.6%), 2–4 (Group B) in 7 patients (31.8%) and >4 (Group C) in 1 patient (4.5%). During the whole period of treatment, Lenvatinib median dose was 14 mg (10–21 mg), while Lenvatinib median starting dose was 16 mg (10–24 mg) (Table 2). Thirteen out of 22 patients (59.1%) underwent a first dose down titration with a median reduction of 4 mg (−10–4 mg) and 5/22 patients (27.8%) underwent a further dose reduction with a median decrease of 2 mg (−4–0). Statistical analysis did not show any association between baseline CONUT score and Lenvatinib starting dose (rs 0.179, *p* = 0.477 at Spearman’s rank correlation test) or any dose reductions (rs 0.230, *p* = 0.450 and rs 0.186, *p* = 0.764 between baseline CONUT score and first and second reductions, respectively).

**Table 2:** Baseline clinical and histopathological features of patients

	Patients (*n* = 22)
Age (median)	68 (41–84)
Gender (%)	
- Female	14 (64%)
- Male	8 (36%)
BMI (median)	26.9 (20.8–33.5)
Hystotype (%)	
- Papillary	10 (45.4)
- Poorly differentiated	2 (9.1)
- Follicular	4 (18.2)
- Oncocytic	6 (27.3)
Median starting dose (mg)	16 (10–24)
Median dose (mg)	14 (10–21)
Previous TKI (%)	1 (4.5)
ECOG (%)	
- 0	12 (54.6)
- 1	9 (40.9)
- 2	1 (4.5)
CONUT (%)	
- 0–1	14 (63.6)
- 2–4	7 (31.8)
- >4	1(4.5)
Best Tumor Response (BTR) (%)^a^	
- CR	0 (0)
- PR	10 (55.6)
- SD	4 (22.2)
- PD	4 (22.2)

^a^Percentage calculated on 18/22 patients

Nineteen out of 22 patients (86.4%) presented at least one adverse event (AE) of any grade. In detail, the AEs were grade 1–2 in 4/22 (18.2%), grade 3–4 in 12/22 (54.5%) and grade 5 in 3/22 (13.6%). Main adverse events were diarrhea (50%), fatigue/asthenia (50%), decreased appetite/weight (45.5%) and hypertension (31.8%). Less frequently proteinuria (9.1%), nausea/vomiting (4.5%), palmar-plantar erythrodysesthesia syndrome (4.5%), headache (4.5%) or arthralgia (4.5%) occurred. Of note, fistulization and venous thromboembolism occurred only in 2 (9.1%) and 1 (4.5%) patient, respectively, and resulted in patients’ death (Table 3). Statistical analysis did not show any correlation between baseline CONUT score and adverse events severity (rs 0.145, *p* = 0.520 at Spearman’s rank correlation test), while it was observed a correlation between baseline ECOG PS and severity of adverse events (rs 0.468, *p* = 0.027). Indeed, patients with higher baseline ECOG PS had a higher probability to experience higher grade AEs.

**Table 3:** Adverse events (AEs) during treatment with Lenvatinib

Adverse events (AEs)	Patients n (%)		
	All AEs n (%)	Grade 3–4 AEs n (%)	Grade 5 AEs n (%)
Diarrhea	11 (50)	4 (18.18)	—
Fatigue or asthenia	11 (50)	4 (18.18)	—
Decreased appetite/weight	10 (45.45)	4 (18.18)	—
Hypertension	7 (31.81)	5 (22.72)	—
Proteinuria	2 (9.09)	2 (9.09)	—
Fistulization	2 (9.09)	—	2 (9.09)
Nausea/Vomiting	1 (4.54)	—	—
Palmar-plantar erythrodysesthesia syndrome	1 (4.54)	1 (4.54)	—
Headache	1 (4.54)	1 (4.54)	—
Arthralgia	1 (4.54)	—	—
Pulmonary embolism	1 (4.54)	—	1 (4.54)

Finally, our data showed an inverse correlation between baseline CONUT score and baseline phase angle (rs 0.544, *p* = 0.044 at Spearman’s rank correlation test).

### Efficacy of lenvatinib treatment

**Fig. 1:**
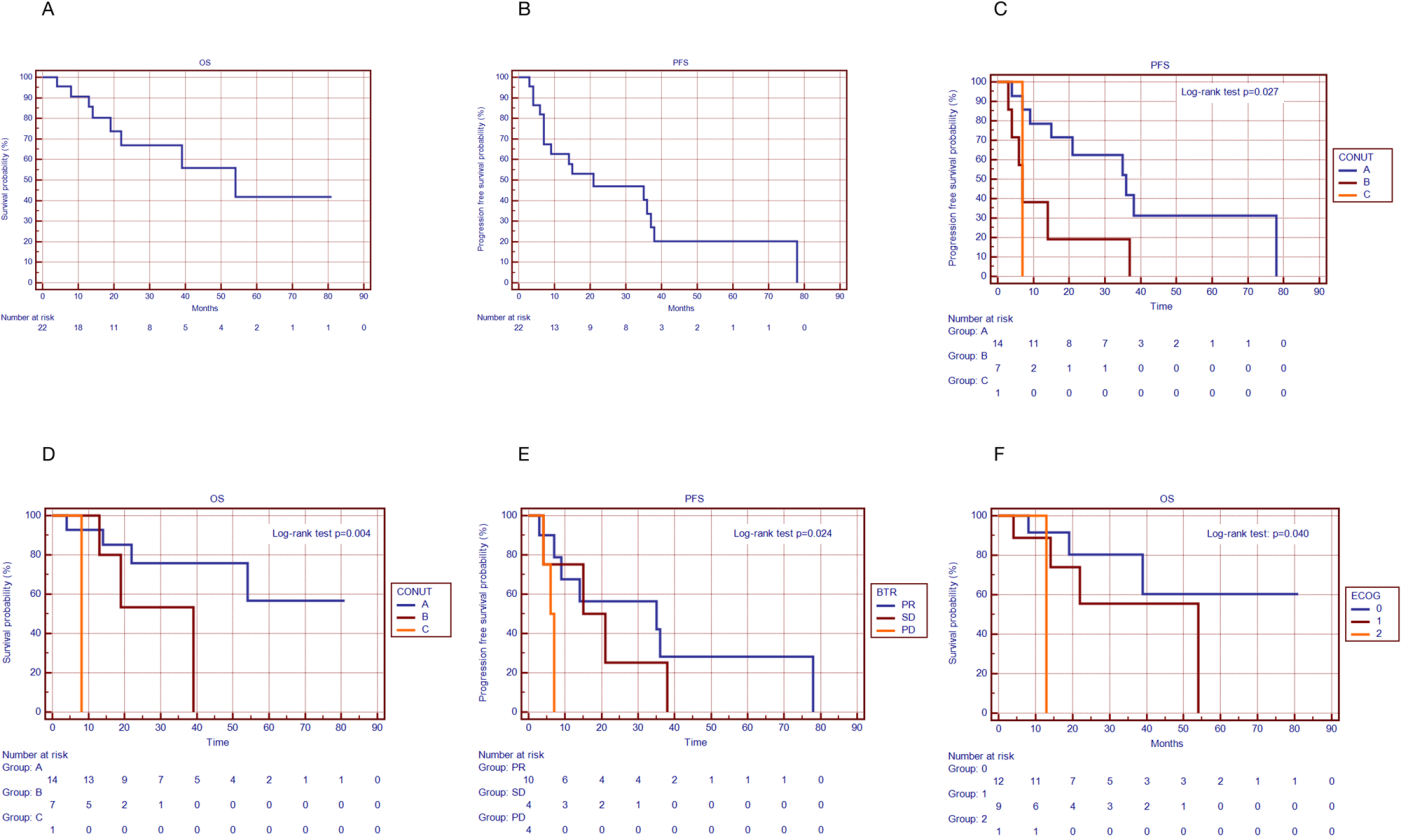
Significant survival curves. **A** Kaplan–Meier curve for OS: Death had occurred in 8 patients (36.4%) with a median overall survival of 54 months (95% CI 20.34–87.65 months). **B** Kaplan–Meier curve for PFS: Progression had occurred in 16 patients (72.7%) with a median PFS of 21 months (95% CI 0–47.33 months). **C** Kaplan–Meier curves for PFS according to CONUT score: Disease progression occurred in 9 of 14 (64.3%) Group A patients with a median PFS of 36 months (95% CI 14.82–57.18 months), in 6 of 7 (85.7%) of Group B patients with a median PFS of 7 months (95% CI 4.94–9.05 months) and in the only 1 patient of Group C, with a PFS of 7 months (Log rank test: *p* = 0.027) (HR 2.77- 95% CI 1.216 - 6.307, *p* = 0.015). **D** Kaplan–Meier curve for OS according to CONUT score: Death occurred in 4 of 14 (28.6%) Group A patients with a median OS that had to be reached and a mean OS of 59.6 (95% CI 42.70–76.55 months), in 3 of 7 (42.9%) Group B patients with a mean OS of 28.47 months (95% CI 15.12–41.82 months)(median OS had yet to be reached) and in the only Group C patient with a survival of 8 months (Log rank test: *p* = 0.004) (HR 4.455 - 95% CI 1.211–16.391, *p* = 0.025). **E** Kaplan–Meier curve for PFS according to Best Tumor Response (BTR): Disease progression occurred in 7 of 10 (70%) patients who got partial response (PR) with a median PFS of 35 months (95% CI 0–85.82 months) and in all patients with stable disease (SD) with a median PFS of 15 months (95% CI 0–31.66 months) and in all the non-responder ones (PD) with a median PFS of 6 months (95% CI 4.04–7.96 months) (Log rank test: *p* = 0.024) (HR 2.337 - 95% CI 1.091–5.006, *p* = 0.029). **F** Kaplan–Meier curve for OS according to ECOG PS: in the ECOG 0 group and ECOG 1 group, 3 of 12 (25%) and 4 of 9 (44.4%) patients, respectively, died with a median OS that had to be reached, and in the ECOG 2 group death occurred in the only patient included with an OS of 13 months (Log-rank test: *p* = 0.040) (HR 3.251 - 95% CI 0.971–10.889, *p* = 0.056)

At the time of analysis, 14 patients (63.6%) were still alive with a median overall survival of 54 months (95% CI 20.3–87.7 months) (Fig. 1A). Progression had occurred in 16 patients (72.7%) with a median PFS of 21 months (95% CI 0–47.33 months) (Fig. 1B). We didn’t find any correlation between the above clinical outcomes and patient’s baseline characteristics except for CONUT score and BTR (Table 4).

**Table 4:** Univariate Cox-regression analysis of PFS and OS

	PFS	OS
Age	*p* = 0.563	*p* = 0.453
CONUT score	***p*** = **0.015**	***p*** = **0.025**
BMI	*p* = 0.433	*p* = 0.888
ECOG PS	*p* = 0.185	*p* = 0.056
Lenvatinib median dose	*p* = 0.392	*p* = 0.261
Best Tumor Response	***p*** = **0.029**	*p* = 0.082

After Lenvatinib initiation, BTR could be detected in 18 out of 22 patients (81.8%). Overall response rate (ORR) was 55.6% with partial response (PR) observed in 10 patients (55.6%), stable disease (SD) in 4 patients (22.2%), and progressive disease (PD) in 4 patients (22.2%), while no patient achieved a complete response (CR) (Table 2). The median tumor burden modification was −30% (range +30 – −63.4%).

### CONUT score predicts clinical outcome

At the time of observation, disease progression occurred in 9 out of 14 (64.3%) Group A patients, in 6 out of 7 (85.7%) Group B patients and in the only 1 patient of Group C. Median PFS was 36 months (95% CI 14.82–57.18 months) in Group A and 7 months (95% CI 4.94–9.05 months) in Group B, while PFS in the Group C patient was 7 months (Log rank test: *p* = 0.027, Fig. 1C). Death occurred in 4 of 14 (28.6%) Group A patients, in 3 of 7 (42.9%) Group B patients and in the only Group C patient. Group A and Group B median OS had yet to be reached so the estimated mean OS was 59.6 months (95% CI 42.71–76.55 months) and 28.5 months (95% CI 15.12–41.82 months) respectively, while Group C median OS was 8 months (Log rank test: *p* = 0.004, Fig. 1D) (Table 5). The univariate Cox-regression analysis revealed an association between CONUT score and both study outcomes, showing a significantly reduced PFS (HR 2.77- 95% CI 1.216–6.307, *p* = 0.015) and OS (HR 4.455 - 95% CI 1.211–16.391, *p* = 0.025) moving from Group A to Group B and from Group B to Group C.

**Table 5:** Response to therapy according to baseline CONUT score

	Patients n (%)	PFS median (months)	OS median (months)
Group A (CONUT 0–1)	14 (63,6%)	36 (14.8–57.2)	59.6^a^ (42.71–76.55)
Group B (CONUT 2–4)	7 (31,8%)	7 (4,94–9,05)	28.5^a^ (15.12–41.82)
Group C (CONUT >4)	1 (4,5%)	7	8

^a^Group A and Group B median OS couldn’t be calculated because more than 50% of patients were still alive at the time of analysis

### Best tumor response (BTR) predicts clinical outcome

Disease progression occurred in 7 of 10 (70%) patients who got partial response (PR) to Lenvatinib therapy and in all patients with stable disease (SD) as well as in all the non-responder ones (PD), with a median PFS of 35 months (95% CI 0–85.82 months), 15 months (95% CI 0–31.66 months) and 6 months (95% CI 4.04–7.96 months), respectively (Log rank test: *p* = 0.024, Fig. 1E). At the univariate Cox-regression analysis, we observed an association between BTR and PFS with a significantly prolonged PFS (HR 2.337 - 95% CI 1.091–5.006, *p* = 0.029) in responder patients compared to those with stable disease and in the latter compared to those with progressive disease.

When CONUT score and BTR entered together in a multivariate Cox regression model with PFS as dependent variable, the significance that they had in univariate analysis was lost (*p* = 0.238 and *p* = 0.261, respectively).

### ECOG PS predicts clinical outcome

Finally, the univariate Cox-regression showed a not significant association between ECOG PS and OS (HR 3.251 - 95% CI 0.971–10.889, *p* = 0.056) but with a significantly longer survival in patients with ECOG 0 compared to the ECOG 1 and in the latter compared to ECOG 2 group (Log-rank test: *p* = 0.040). In the ECOG 0 group, 3 of 12 (25%) patients died with a mean OS of 59.4 months (95% CI 39.43–79.34 months) (median OS hadn’t been reached). Conversely, in the ECOG 1 group death occurred in 4 of 9 (44.4%) patients with a mean OS of 36.6 months (95% CI 19.01–54.09 months) (median OS hadn’t been reached). Finally, in the ECOG 2 group death occurred in the only patient included with an OS of 13 months (Fig. 1F).

## Discussion

DTC accounts for about 95% of thyroid tumors and it mostly shows a favorable outcome. In those rare patients suffering from progressive RAI-R-DTC, systemic therapy with MKIs has proven to be effective. Efficacy of the MKI Lenvatinib was demonstrated in the multicenter, randomized, double blind SELECT study [3]. Once approved, Lenvatinib efficacy in progressive RAI-R-DTC was confirmed by several real-life studies. However, most of the initial experiences showed global inferior results if compared with SELECT study and this may be explained by worse clinical baseline characteristics, more advanced disease stages and previous systemic treatments of real-life patients [6, 18]. Our study reported a median PFS of 21 months in RAI-R-DTC patients treated with Lenvatinib. It was longer than the one reported in other real-life studies but also longer than the SELECT one. Some baseline characteristics of our study population can partially explain these results: (a) the use of Lenvatinib as fist-line systemic therapy in all but one patient, (b) the good baseline performance status and (c) the good baseline immuno-nutritional status. A subanalysis of the SELECT study showed a median PFS of 18.7 months in TKI-naïve patients, longer than the median PFS of 15.1 months recorded in patients previously treated with another TKI [3]. Furthermore, a longer PFS could also be related to the prompt start of Lenvatinib at the documented progression and before clinical worsening: 21 of 22 (95.45%) patients had, in fact, a baseline ECOG PS of 0 or 1 [19]. Despite an overall baseline good performance status, we observed that patients with a baseline ECOG PS of 0 had an improved OS compared to patients with an ECOG PS of 1 with the latter having a better OS compared to the ECOG PS 2 group. These results are consistent with the ones reported in a post hoc analysis of SELECT study that assessed ECOG PS as prognostic marker in RAI-R-DTC patients treated with Lenvatinib showing a longer OS (HR 0.42 [95% CI 0.26–0.69]; *p* = 0.0004) in patients with baseline ECOG PS of 0, compared to ECOG PS of 1 [20]. Conversely, in our study higher baseline ECOG PS correlated with higher severity of AEs. Notably, the recorded AEs were superimposable to those of other similar series.

Moreover, consistently with SELECT results and real-life studies, partial response (PR) was the response that occurred more frequently among our study population. We observed a median PFS of 35 months in patients who responded to Lenvatinib treatment with a decrease in tumor size >30% (no patient obtained a complete response) compared to 21 and 6 months in the SD or PR patient groups, respectively. These data are similar to those observed in the responder cohort of patients treated with Lenvatinib in the SELECT study [4].

Finally, also our patients’ better immuno-nutritional status may have contributed to the more favorable outcomes. Indeed, data from our patients’ baseline bioelectrical impedance showed a median phase angle >5, identifying a quite satisfactory patients’ body composition given the underlying cancer. Disease related malnutrition is highly prevalent among cancer patients during the course of disease and can negatively affect treatment tolerance and consequent response to therapy [9, 21, 22]. Thus, the need to perform an early nutritional screening in order to prevent or to treat the consequences of cancer-related malnutrition is evident [8]. Several nutritional scoring systems, such as Nutritional Risk Screening (NRS), albumin (ALB), and Prognostic Nutritional Index (PNI), proved to be prognostic factors in cancer patients [23, 24]. In this setting, the Controlling Nutritional Status (CONUT) has been shown to predict prognosis in several types of tumors and was assessed as prognostic factor for OS in different solid cancers [25]. Because it includes cholesterol in addiction to lymphocyte count and albumin, it better reflects immune and nutritional status of patients. Albumin is a marker of systemic inflammation [26]. Indeed, hypoalbuminemia correlates with increased levels of multiple inflammatory factors, such as interleukin-6 (IL-6) and tumor necrosis factor alpha (TNF-α), leading to tumor inflammation progression [27]. Furthermore, the total lymphocyte count is a marker of impaired immune defenses due to malnutrition and cholesterol levels strongly correlates with tumors proliferation in different ways: (a) by reducing T cell depletion in tumor microenvironment; (b) by regulating the innate and adaptive immune responses of multiple immune cells in tumors [28–30].

Despite its validation as a prognostic factor in other Lenvatinib-treated cancers, Dalmiglio et. al firstly assessed CONUT score in TKI-treated advanced thyroid cancer patients showing a significant association between baseline CONUT score and PFS [HR 12.211 (95% CI 4.084–36.512), *p* < 0.0001] and between baseline CONUT score and OS [HR 23.551 (95% CI 4.949–112.060), *p* < 0.0001] [16]. However, they included patients with multiple histotypes of thyroid cancer (including medullary thyroid carcinoma) and treated with different TKIs. Instead, in our study we assessed the prognostic value of CONUT score in a selected population of RAI-R-DTC treated with Lenvatinib as first line systemic therapy in all but one case. By computing CONUT score in its original description, we mostly observed a normal nutritional status or light malnutrition (Group A and B), with only one patient affected by moderate/severe malnutrition (Group C) before Lenvatinib start. Furthermore, as expected, we observed that patients with worse immune-nutritional status (higher baseline CONUT score) had a worse body composition (reduced phase angle), resulting in a correlation between biochemical parameters and bioelectrical impedance analysis.

Our data proved the prognostic value of CONUT score in terms of response to Lenvatinib therapy. Indeed, we observed an association between baseline CONUT score and clinical outcomes, showing a significantly reduced PFS and OS in patients with baseline higher CONUT score (Group B and Group C). Furthermore, the baseline CONUT score did not affect either the initial dosage of Lenvatinib or dose reductions. Thus, we could exclude a bias driven by the use of lower Lenvatinib doses in patients with a worse nutritional status. The above results suggest a potential role of nutritional status in clinical outcomes of patients with advanced thyroid cancer undergoing Lenvatinib therapy and CONUT score represents a potential screening tool to identify and eventually to correct malnutrition before Lenvatinib initiation.

Possible limitation of this study is the loss of statistical significance of the associations in multivariate analyses. I.e. association between CONUT score and BTR with PFS was lost at multivariate Cox-regression analysis. Nevertheless, the lack of independent association of the considered variables with the outcome may be explained by their strict interdependency. I.e., a nearly significant association between CONUT score and BTR was found at nonparametric correlation (data not shown) and by the relatively small sample size.

## Conclusion

Lenvatinib is currently the first-line systemic therapy in progressive radioiodine-refractory thyroid cancer. Our data confirm its efficacy in terms of PFS and OS showing more favorable outcome when promptly started at the documented progression. Early initiation of therapy prevents disease-related worsening of clinical conditions ensuring better tolerability to therapy and fewer side effects. Since therapeutic response may, also, be impaired by disease-related malnutrition, the latter should be evaluated at Lenvatinib start and CONUT score represents an easily applicable malnutrition screening tool that showed a prognostic value in this setting. Therefore, the improvement of baseline nutritional status and physical performance with dedicated prehabilitation programs could determine better drug efficacy and disease prognosis.

## References

[1] L. Fugazzola, R. Elisei, D. Fuhrer, B. Jarzab, S. Leboulleux, K. Newbold, J. Smit, 2019 European thyroid association guidelines for the treatment and follow-up of advanced radioiodine-refractory thyroid cancer. Eur. Thyroid. J. **8**, 227–245 (2019). 31768334 10.1159/000502229 PMC6873012

[2] Z. Hussein, H. Mizuo, S. Hayato, M. Namiki, R. Shumaker, Clinical Pharmacokinetic and pharmacodynamic profile of lenvatinib, an orally active, small-molecule, multitargeted tyrosine kinase inhibitor. Eur. J. Drug. Metab. Pharmacokinet. **42**, 903–914 (2017). 28236116 10.1007/s13318-017-0403-4

[3] M. Schlumberger, M. Tahara, L.J. Wirth, B. Robinson, M.S. Brose, R. Elisei, M.A. Habra, K. Newbold, M.H. Shah, A.O. Hoff, A.G. Gianoukakis, N. Kiyota, M.H. Taylor, S.B. Kim, M.K. Krzyzanowska, C.E. Dutcus, B. de las Heras, J. Zhu, S.I. Sherman, Lenvatinib versus placebo in radioiodine-refractory thyroid cancer. N. Engl. J. Med. **372**, 621–630 (2015). 25671254 10.1056/NEJMoa1406470

[4] A.G. Gianoukakis, C.E. Dutcus, N. Batty, M. Guo, M. Baig, Prolonged duration of response in lenvatinib responders with thyroid cancer. Endocr. Relat. Cancer **25**, 699–704 (2018). 29752332 10.1530/ERC-18-0049 PMC5958278

[5] R.I. Haddad, M. Schlumberger, L.J. Wirth, E.J. Sherman, M.H. Shah, B. Robinson, C.E. Dutcus, A. Teng, A.G. Gianoukakis, S.I. Sherman, Incidence and timing of common adverse events in Lenvatinib-treated patients from the SELECT trial and their association with survival outcomes. Endocrine **56**, 121–128 (2017). 28155175 10.1007/s12020-017-1233-5 PMC5368192

[6] A. Berdelou, I. Borget, Y. Godbert, T. Nguyen, M.E. Garcia, C.N. Chougnet, A. Ferru, C. Buffet, O. Chabre, O. Huillard, S. Leboulleux, M. Schlumberger, Lenvatinib for the treatment of radioiodine-refractory thyroid cancer in real-life practice. Thyroid **28**, 72–78 (2018). 29048237 10.1089/thy.2017.0205

[7] C. Balmelli, N. Railic, M. Siano, K. Feuerlein, R. Cathomas, V. Cristina, C. Güthner, S. Zimmermann, S. Weidner, M. Pless, F. Stenner, S.I. Rothschild, Lenvatinib in advanced radioiodine-refractory thyroid cancer - a retrospective analysis of the swiss lenvatinib named patient program. J. Cancer **9**, 250–255 (2018). 29344270 10.7150/jca.22318 PMC5771331

[8] L. Bargetzi, C. Brack, J. Herrmann, A. Bargetzi, L. Hersberger, M. Bargetzi, N. Kaegi-Braun, P. Tribolet, F. Gomes, C. Hoess, V. Pavlicek, S. Bilz, S. Sigrist, M. Brändle, C. Henzen, R. Thomann, J. Rutishauser, D. Aujesky, N. Rodondi, J. Donzé, A. Laviano, Z. Stanga, B. Mueller, P. Schuetz, Nutritional support during the hospital stay reduces mortality in patients with different types of cancers: secondary analysis of a prospective randomized trial. Ann. Oncol. **32**, 1025–1033 (2021). 34022376 10.1016/j.annonc.2021.05.793

[9] E. Reber, K.A. Schönenberger, M.F. Vasiloglou, Z. Stanga, Nutritional risk screening in cancer patients: the first step toward better clinical outcome. Front. Nutr. **8**, 603936 (2021). 33898493 10.3389/fnut.2021.603936 PMC8058175

[10] J. Ignacio de Ulíbarri, A. González-Madroño, N.G. de Villar, P. González, B. González, A. Mancha, F. Rodríguez, G. Fernández, CONUT: a tool for controlling nutritional status. First validation in a hospital population. Nutr. Hosp. **20**, 38–45 (2005). 15762418

[11] T. Toyokawa, N. Kubo, T. Tamura, K. Sakurai, R. Amano, H. Tanaka, K. Muguruma, M. Yashiro, K. Hirakawa, M. Ohira, The pretreatment Controlling Nutritional Status (CONUT) score is an independent prognostic factor in patients with resectable thoracic esophageal squamous cell carcinoma: results from a retrospective study. BMC Cancer **16**, 722 (2016). 27599460 10.1186/s12885-016-2696-0 PMC5013653

[12] J. Ni, Y. Fang, J. Zhang, X. Chen, Predicting prognosis of heart failure using common malnutrition assessment tools: a systematic review and meta-analysis. Scott. Med. J. **67**, 157–170 (2022). 36052423 10.1177/00369330221122300

[13] K. Fukushima, Y. Ueno, N. Kawagishi, Y. Kondo, J. Inoue, E. Kakazu, M. Ninomiya, Y. Wakui, N. Saito, S. Satomi, T. Shimosegawa, The nutritional index ‘CONUT’ is useful for predicting long-term prognosis of patients with end-stage liver diseases. Tohoku J. Exp. Med. **224**, 215–219 (2011). 21701127 10.1620/tjem.224.215

[14] J.I. de Ulíbarri Pérez, A. González-Madroño Giménez, P. González Pérez, G. Fernández, F. Rodríguez Salvanés, A. Mancha Alvarez-Estrada, A. Díaz, Nuevo procedimiento para la detección precoz y control de la desnutrición hospitalaria [New procedure for the early detection and control of hospital malnutrition]. Nutr. Hosp. **17**, 179–188 (2002). 12395607

[15] J. Chen, P. Song, Z. Peng, Z. Liu, L. Yang, L. Wang, J. Zhou, Q. Dong, The controlling nutritional status (CONUT) score and prognosis in malignant tumors: a systematic review and meta-analysis. Nutr. Cancer **74**, 3146–3163 (2022). 35382655 10.1080/01635581.2022.2059091

[16] C. Dalmiglio, L. Brilli, M. Campanile, C. Ciuoli, A. Cartocci, M.G. Castagna, CONUT score: a new tool for predicting prognosis in patients with advanced thyroid cancer treated with TKI. Cancers **14**, 724 (2022). 35158991 10.3390/cancers14030724 PMC8833681

[17] E.A. Eisenhauer, P. Therasse, J. Bogaerts, L.H. Schwartz, D. Sargent, R. Ford, J. Dancey, S. Arbuck, S. Gwyther, M. Mooney, L. Rubinstein, L. Shankar, L. Dodd, R. Kaplan, D. Lacombe, J. Verweij, New response evaluation criteria in solid tumours: revised RECIST guideline (version 1.1). Eur. J. Cancer **45**, 228–247 (2009). 19097774 10.1016/j.ejca.2008.10.026

[18] L.D. Locati, A. Piovesan, C. Durante, M. Bregni, M.G. Castagna, S. Zovato, M. Giusti, T. Ibrahim, E. Puxeddu, G. Fedele, G. Pellegriti, G. Rinaldi, D. Giuffrida, F. Verderame, F. Bertolini, C. Bergamini, A. Nervo, G. Grani, S. Rizzati, S. Morelli, I. Puliafito, R. Elisei, Real-world efficacy and safety of lenvatinib: data from a compassionate use in the treatment of radioactive iodine-refractory differentiated thyroid cancer patients in Italy. Eur. J. Cancer **118**, 35–40 (2019). 31299580 10.1016/j.ejca.2019.05.031

[19] S. De Leo, M. Di Stefano, L. Persani, L. Fugazzola, C. Colombo, Lenvatinib as first-line treatment for advanced thyroid cancer: long progression-free survival. Endocrine **72**, 462–469 (2021). 32885329 10.1007/s12020-020-02477-0

[20] M.H. Taylor, S. Takahashi, J. Capdevila, M. Tahara, S. Leboulleux, N. Kiyota, C.E. Dutcus, R. Xie, B. Robinson, S. Sherman, M.A. Habra, R. Elisei, L.J. Wirth, Correlation of performance status and neutrophil-lymphocyte ratio with efficacy in radioiodine-refractory differentiated thyroid cancer treated with lenvatinib. Thyroid **31**, 1226–1234 (2021). 33637020 10.1089/thy.2020.0779 PMC8377516

[21] M. Muscaritoli, S. Lucia, A. Farcomeni, V. Lorusso, V. Saracino, C. Barone, F. Plastino, S. Gori, R. Magarotto, G. Carteni, B. Chiurazzi, I. Pavese, L. Marchetti, V. Zagonel, E. Bergo, G. Tonini, M. Imperatori, C. Iacono, L. Maiorana, C. Pinto, D. Rubino, L. Cavanna, R. Di Cicilia, T. Gamucci, S. Quadrini, S. Palazzo, S. Minardi, M. Merlano, G. Colucci, P. Marchetti, PreMiO study group. prevalence of malnutrition in patients at first medical oncology visit: the PreMiO study. Oncotarget **8**, 79884–79896 (2017). 29108370 10.18632/oncotarget.20168 PMC5668103

[22] W.D. Dewys, C. Begg, P.T. Lavin, P.R. Band, J.M. Bennett, J.R. Bertino, M.H. Cohen, H.O. Douglass Jr, P.F. Engstrom, E.Z. Ezdinli, J. Horton, G.J. Johnson, C.G. Moertel, M.M. Oken, C. Perlia, C. Rosenbaum, M.N. Silverstein, R.T. Skeel, R.W. Sponzo, D.C. Tormey, Prognostic effect of weight loss prior to chemotherapy in cancer patients. East. Cooperative Oncol. Group. Am. J. Med. **69**, 491–497 (1980). 10.1016/s0149-2918(05)80001-3 7424938

[23] Y. Zang, W. Xu, Y. Qiu, D. Gong, Y. Fan, Association between risk of malnutrition defined by the nutritional risk screening 2002 and postoperative complications and overall survival in patients with cancer: a meta-analysis. Nutr. Cancer **75**, 1600–1609 (2023). 37382336 10.1080/01635581.2023.2227402

[24] X. Wang, Y. Wang, The prognostic nutritional index is prognostic factor of gynecological cancer: a systematic review and meta-analysis. Int. J. Surg. **67**, 79–86 (2019). 31185310 10.1016/j.ijsu.2019.05.018

[25] S. Kheirouri, M. Alizadeh, Prognostic potential of the preoperative controlling nutritional status (CONUT) score in predicting survival of patients with cancer: a systematic review. Adv. Nutr. **12**, 234–250 (2021). 32910812 10.1093/advances/nmaa102 PMC7850023

[26] E. Gremese, D. Bruno, V. Varriano, S. Perniola, L. Petricca, G. Ferraccioli, Serum albumin levels: a biomarker to be repurposed in different disease settings in clinical practice. J. Clin. Med. **12**, 6017 (2023). 37762957 10.3390/jcm12186017 PMC10532125

[27] A. Eckart, T. Struja, A. Kutz, A. Baumgartner, T. Baumgartner, S. Zurfluh, O. Neeser, A. Huber, Z. Stanga, B. Mueller, P. Schuetz, Relationship of nutritional status, inflammation, and serum albumin levels during acute illness: a prospective study. Am. J. Med. **133**, 713–722.e7 (2020). 31751531 10.1016/j.amjmed.2019.10.031

[28] P. Zhou, B. Li, B. Liu, T. Chen, J. Xiao, Prognostic role of serum total cholesterol and high-density lipoprotein cholesterol in cancer survivors: a systematic review and meta-analysis. Clin. Chim. Acta **477**, 94–104 (2018). 29223765 10.1016/j.cca.2017.11.039

[29] B. Huang, B.L. Song, C. Xu, Cholesterol metabolism in cancer: mechanisms and therapeutic opportunities. Nat. Metab. **2**, 132–141 (2020). 32694690 10.1038/s42255-020-0174-0

[30] X. Ma, E. Bi, Y. Lu, P. Su, C. Huang, L. Liu, Q. Wang, M. Yang, M.F. Kalady, J. Qian, A. Zhang, A.A. Gupte, D.J. Hamilton, C. Zheng, Q. Yi, Cholesterol induces CD8+ T cell exhaustion in the tumor microenvironment. Cell Metab. **30**, 143–156.e5 (2019). 31031094 10.1016/j.cmet.2019.04.002 PMC7061417

